# Treatment Strategies and Complications in Reverse-Oblique Trochanteric Femur Fractures and Evaluation of a New Classification System

**DOI:** 10.3390/jcm15041502

**Published:** 2026-02-14

**Authors:** Robert Breuer, Rainer Fiala, Theresa Dorner, Barbara Strasser-Kirchweger, Harald Kurt Widhalm, Mehdi Mousavi, Nikolaus Wilhelm Lang

**Affiliations:** 1 Department of Orthopedics and Trauma Surgery, Klinik Donaustadt, Langobardenstraße 122, 1220 Vienna, Austria; rainer.fiala@franziskusspital.at (R.F.); theresa.dorner@gesundheitsverbund.at (T.D.); mehdi.mousavi@gesundheitsverbund.at (M.M.); 2 Department of Psychology, Centre for Cognitive Neuroscience, University of Salzburg, Hellbrunnerstraße 34, 5020 Salzburg, Austria; barbara.strasser-kirchweger@plus.ac.at; 3 Department of Orthopedics and Trauma Surgery, Medical University of Vienna, Waehringer Guertel 18-20, 1090 Vienna, Austria; harald.widhalm@meduniwien.ac.at (H.K.W.); nikolaus.lang@meduniwien.ac.at (N.W.L.)

**Keywords:** reverse-oblique fracture, femoral fracture, cephalomedullary implant, classification reverse-oblique fracture, open/closed reduction

## Abstract

**Background:** Reverse-oblique femoral fractures are regarded as highly unstable and are still associated with high complication and failure rates. A new classification system is said to facilitate risk assessment and decision-making. **Methods:** Over ten years, 7804 patients with per/subtrochanteric fractures were screened in this retrospective analysis. A total of 552 patients with a reverse-oblique fracture pattern were included. The fractures were classified according to the new classification system. The choice of implants, complication rates, revision surgery, and time of surgery were recorded. Radiological outcome parameters and dislocation were measured. **Results:** For the classification, a good intra-rater reliability (r = 0.77) and inter-rater reliability (k = 0.64) were calculated. The complication rate was overall 19% (n = 105). More than 60% of complications needed revision surgery. The most common complications were cut-out and implant failure (3%); only Parker’s ratio, as a radiological parameter, had prognostic value. Malreduction had a negative impact on mal- or non-unions (*p* < 0.01), and a trend towards higher overall complications (*p* = 0.52). Prolonged time of surgery increased the overall complication rate (r = 0.2, *p* < 0.001). The same was found after open reduction (*p* = 0.005, OR 2.00). The use of cerclage wires had no positive or negative effects. The use of short or long implants did not influence the outcome. **Conclusions:** Reverse-oblique femoral fractures are associated with a high complication rate. Short implants can be safely used in cases without severe dislocation if a sufficient working length is considered. Anatomical reduction benefits the outcome as long as it can be performed closed. The classification system presents good inter- and intra-rater reliability.

## 1. Introduction

The incidence of proximal femoral fractures, including intertrochanteric fractures, is constantly increasing in post-industrialized societies due to the aging population [1,2]. It is expected that the number of patients with this type of injury will reach 6.3 million per year by 2050, which will cause an enormous burden on health care systems [3,4]. Most importantly, the impact on every single patient can be severe, with noticeable and often permanent impairment of mobility and, consequently, activities of daily living, independence, as well as subsequent high mortality rates of up to 20% per year [5,6]. The reverse-oblique fracture type (AO/OTA 31A-3) accounts for about 5% of all inter- and subtrochanteric fractures [7]. The risk of catastrophic failure and other surgical complications is high due to its inherent instability [7,8,9]. Instability results from the fracture morphology, which is characterized by a fracture line between the attachment area of the medial and lateral bundle of the iliofemoral ligament and the lack of medial buttress support [10]; therefore, the fragments are regularly dislocated medially and proximally due to traction of the adductor and iliopsoas muscles [11]. Treatment with cephalomedullary implants is standard in this fracture type, showing superior outcomes clinically and radiologically and reducing the necessity of revision surgery when compared to plates or sliding hip screws [12,13,14,15,16]. The main benefit is the intramedullary buttress effect, which prevents shaft medicalization as the common mode of failure [3]. Routinely, long implants are used in the treatment of subtrochanteric fracture, without clear evidence of superiority for long cephalomedullary nails [17,18,19]. Biomechanical studies still cannot provide clear cut-off values for choice of implant length [3,19]. However, prolonged surgery time, soft tissue damage, or increased blood loss when using long implants are of high clinical relevance [17,18,20,21]. Certainly, the lack of a comprehensive classification system, which can give clear treatment algorithms, is part of the problem. In the current literature, only two studies introduced a classification system for this special fracture type [10,22]. The aim of this study was to evaluate a previously published classification system for reverse-oblique femoral fractures, determine its use for clinical- or research-related decision-making and comparability, and furthermore shed light on the question of the choice of implants and possible drawbacks.

## 2. Materials and Methods

Over a ten-year time period, the databases of two major trauma centers were screened for any patients treated with a pertrochanteric/subtrochanteric femoral fracture. Inclusion criteria comprised all forms of reverse-oblique intertrochanteric fracture patterns (AO/OTA 31A-3), low-impact trauma mechanism (simple falls from a standing position), treatment with a long or short cephalomedullary implant (Proximal Femoral Nail Antirotation, Synthes, West Chester, PA, USA; Gamma Nail, Stryker, Kalamazoo, MI, USA), with or without cerclage wires, acute fractures (time from injury to surgery <2 weeks). A minimum follow-up of 12 months was mandatory. Exclusion criteria were sole subtrochanteric fractures with the medial fracture line beginning distally of the lesser trochanter, due to different biomechanical properties [23], pathological fractures and high-velocity trauma mechanism, polytraumatized patients (ISS > 16), or treatment via different implant. This study was approved by the local ethics committee (Ethical vote number: EK-23-059-VK) and carried out according to the Declaration of Helsinki.

### 2.1. Radiological Assessment

**Figure 1:**
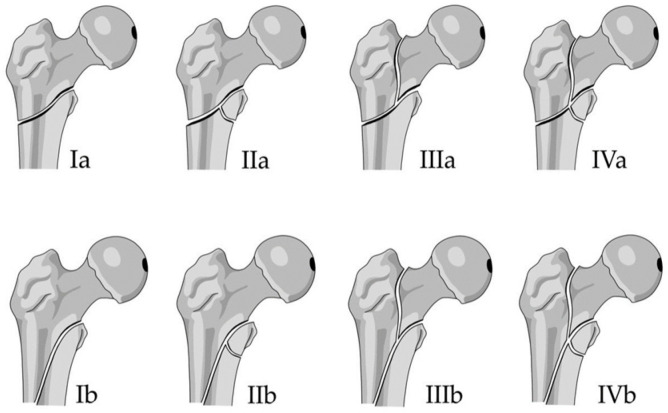
Classification of reverse-oblique femoral fractures (schematics): Fractures subdivided into four main groups (I–IV) based on rate of comminution, which are subdivided into two subgroups a and b, according to the height of the lateral fracture line. Figure 1 shows the schematic.

**Figure 2:**
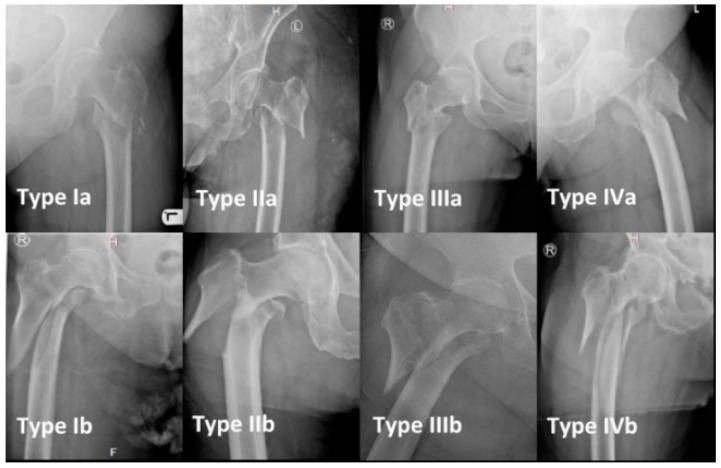
Classification of reverse-oblique femoral fractures (radiographs): Fractures subdivided into four main groups (I–IV) based on rate of comminution, which are subdivided into two subgroups a and b, according to the height of the lateral fracture line. Figure 2 shows the respective radiographs (R = right side, L = left side, H = head/cranial, F = foot/caudal).

All preoperative radiographs of all operated patients were screened for reverse-oblique fracture patterns (AO/OTA 31A-3) by three different authors. The X-rays of each included patient were re-evaluated by the three authors independently and assigned to the respective classification subgroup according to the previously mentioned classification system. The classification includes four main types (Type I–IV), each subdivided into two subtypes (A and B) [22]. A graphic representation of the classification system is shown in Figure 1 and the respective radiological depiction in Figure 2. If disagreement over a certain case occurred, all the authors were asked to cross-check the X-rays, and as a result, the fracture was classified following discussion and consent. For validation, inter-rater and intra-rater reliability were calculated for the used classification system.

### 2.2. Surgical Technique and Postoperative Regimen

The implantation of the cephalomedullary implants was performed according to standard procedure of each of the two participating trauma centers, with the patient in a supine position on a traction table. The sole difference was the usage of either one C-arm or two C-arms simultaneously for intra-operative verification. The optimal position of the lag screw was determined to be in the center or caudal third of the a.p. plane and in the center of the femoral head in the axial plane. The choice of long or short implant, whether open or closed reduction had to be performed, or whether cerclage wires had to be used, was the sole decision of the operating surgeon. Gamma Nail or PFNA could be chosen as possible implants. The patients were allowed full weight bearing starting on the first postoperative day, using crutches or a wheeled walker. Mobilization was supervised and guided by experienced physiotherapists. Postoperative X-rays were taken on the first postoperative day.

### 2.3. Outcome Parameters

Migration of the lag screw, rotation of the femoral head, and generally the placement of the lag screw were assessed via Parker’s ratio [22]. The tip-apex distance (TAD) and the calcar-referenced tip-apex distance (calTAD) were used to estimate the probability of cut-out and concomitant varus collapse [24,25]. Aforementioned radiographic outcome parameters were measured at the first postoperative X-rays and at the latest follow-up. Malreduction was assessed at the first postoperative a.p. radiograph as well and defined as dislocation of more than 0.5 cm of the medial or lateral cortex. A defective area was defined as contact loss of the medial or lateral corticalis with a width over 0.5 cm. The radiographic measurements were performed by at least three authors independently. The ASA score was used to assess patients’ morbidity. Mean operation time and mean hospital stay were calculated.

### 2.4. Complications

All surgery-related complications were reported, such as migration or lag-screw cut-out and varus collapse, lateralization, implant failure, or loosening. All cases of revision surgery were highlighted as well. Additionally, all patient-related complications that occurred during the hospital stay or could be related to the operation were documented, including infections, intra-operative or related postoperative death, thrombosis, or intra-operative events (e.g., cardiovascular events).

### 2.5. Statistical Analyses

All statistical analyses were undertaken using R (Version 2023.03.0 Build 386) and written as RMarkdown report. The level of significance for all conducted analyses was set to α ≤ 0.05. To validate the classification, both inter-rater and intra-rater reliability were evaluated using a two-way random effect model. The model was applied with a single rater unit assessing agreement. For the calculation of correlation scores, Fisher’s transformation was applied when means were used to assess reliability. For inter-rater reliability, Cohen’s Kappa was employed. Associations between continuous predictors (age, duration of surgery, and radiological measurements) and binary variables (implant length [short vs. long] and complication outcomes [mechanical and non-mechanical complications, yes/no]) were initially explored using point-biserial correlations (Pearson’s product–moment correlation with a dichotomous variable). In the treatment strategy section, associations between age and duration of surgery with implant length were assessed using point-biserial correlations. Variables showing evidence of association with complication outcomes in these exploratory analyses were subsequently entered into multivariable logistic regression models. Implant length was included as a covariate to account for potential confounding related to treatment selection. In addition, a multivariable logistic regression model was constructed to examine the association of age, gender, duration of surgery, and implant length (short vs. long) with the occurrence of mechanical and non-mechanical complications. This model allowed simultaneous adjustment for all included predictors, providing estimates of the independent association of each factor with complication risk. In the exploratory section, a correlation was used for the association of mortality and surgery time. For associations involving only dichotomous variables, Fisher’s exact test was applied to determine the statistical significance of the relationship between two categorical variables. This approach was taken for the comparisons of complications between the subclasses A and B, the associations between complications/treatment strategies and gender, as well as for the assessment of the association between mortality and the classification/treatment strategies. Comparisons of means were performed via an analysis of variance (ANOVA) since the reduction type of the treatment strategies showed three levels, and ANOVA was used to assess the mean differences for age and surgery duration. In the exploratory section, mean differences in surgery duration and radiological parameters across the different complications were assessed using an ANOVA.

## 3. Results

A total of 7804 patients with per/subtrochanteric femoral fractures were screened for eligibility. A total of 552 (7%) patients with a reverse-oblique fracture patternwere included in the final analysis. Fifteen patients had to be excluded because of treatment via implants other than cephalomedullary nails, pathological fractures, or high-velocity trauma. As expected, the population was mostly geriatric, with a mean age of 79 years (±12.2) at the time of surgery. The majority of patients were female, with 452 vs. 100 (82% vs. 18%), which seems to be a similar ratio compared to other trochanteric fracture patterns and highlights the association with osteoporosis [26,27].

### 3.1. Classification

All fractures were classified according to the classification system used. The most prevalent fracture patterns were II and IV. By far, the majority of patients were treated via long implant. An overview concerning the distribution of fracture patterns and used implants can be seen in Table 1. A moderate-to-good intra-rater reliability r = 0.77 (O1: r = 0.65; O2: r = 0.88) and a substantial inter-rater reliability k = 0.64 (T1: k = 0.61; T2: k = 0.67) was calculated for the previously mentioned classification system.

### 3.2. Complications

In 105 patients (19%), complications related to the surgery were detected. In 65 patients (11% of all patients, 62% of registered complications), revision surgery was needed. The most frequent complications found were nail cut-out in 21 patients, implant failure in 18 patients, followed by wound infections in 15 patients and lateral migration of the screw in 10 patients.

The list with all noted complications, numbers, and percentages can be seen in Table 2. The one-year mortality rate was at 25% (n = 136). The fracture type did not have any significant influence on the one-year mortality rate (*p* = 0.3581, OR 0.4–1, CI 95%). No increased mortality could be found in revision cases either (*p* > 0.05).

### 3.3. Influencing Factors on Complications

Fracture classification did not have any predictive value regarding outcome or overall complication rate and mechanical complications in detail. This applies to both the main classification, Types I–IV, and subclassification, A and B (*p* = 0.637).

Long implants were chosen in the majority of the cases (long: n = 407 vs. short: n = 145; *p* ≤ 0.05). To account for potential selection bias related to implant length, we included implant length as a covariate in the multivariable logistic regression model examining factors influencing complications, if it was significant in the initial unadjusted correlations. The chosen implant did not have any influence on the complication rate in Type A or Type B fractures (*p* > 0.05). When looking at implant-related complications alone (nail breakage, lateral migration of the screw/blade, or aseptic loosening), no influence related to the choice of implant could be observed either (*p* > 0.05). As expected, the implantation of long nails increased time of surgery significantly (r = 0.35, *p* < 0.001).

**Figure 3:**
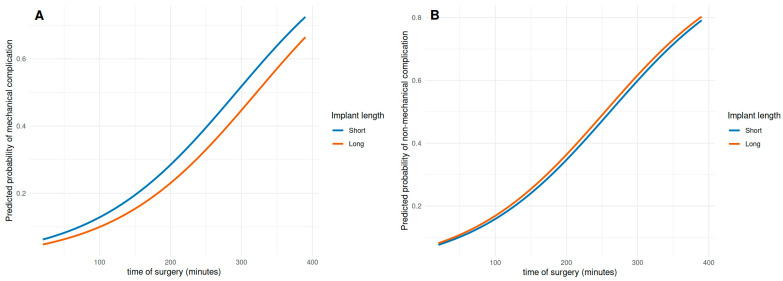
Association between time of surgery and complication rate: increased time of surgery is associated with an increase in mechanical (**A**) and non-mechanical complications (**B**), without being influenced by the choice of implant.

An overall higher complication rate was found in cases where open reduction was deemed necessary (χ^2^ = 7.442, *p* = 0.005, OR 2.00). When analyzing the impact on commonly associated complications like avascular necrosis or non-union, no correlation could be found (*p* > 0.05). As expected, in cases with open reduction, time of surgery increased significantly (68 min vs. 118 min, *p* < 0.05). Increased time of surgery, in turn, increased the overall complication rate (r = 0.2, *p* < 0.001). In the logistic regression model too, time of surgery was significantly associated with mechanical (OR = 1.01, *p* < 0.001) and non-mechanical (OR = 1.01, *p* < 0.001) complication rates, indicating that each additional minute of operation time increased the odds of a complication. In contrast, the implant length alone was not significantly associated with complication rate in mechanical (OR = 0.75, *p* = 0.433) or non-mechanical complications (OR = 1.08, *p* = 0.82). The model showed an improvement in fit compared to the null model in mechanical (null deviance = 335.31, residual deviance = 324.52, AIC = 330.52) and non-mechanical (null deviance = 445.36, residual deviance = 428.07, AIC = 434.07) complication rates. The regression model is depicted in Figure 3A,B.

There was no significant influence of increased surgical time on mortality rate (*p* > 0.05).

**Figure 4:**
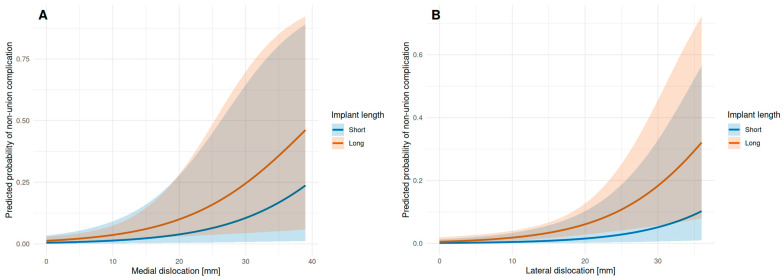
Influence of medial/lateral dislocation on non-union rate: Medial (**A**) as well as lateral (**B**) malreduction have a significant influence on rate of non-unions, independent of the choice of implant. In the corresponding figure showing predicted probabilities with 95% confidence intervals (shaded areas), the overlapping shaded bands for long vs. short implants illustrate that the difference between implant lengths is not statistically significant, even though the point estimate lines appear separated.

Postoperative malreduction in the medial or lateral corticalis significantly increased the rate of mal- or non-union of the fracture (*p* < 0.01). In the logistic regression model, both medial (OR = 1.11, *p* = 0.00652) and lateral (OR = 1.13, *p* < 0.001) dislocations were significantly associated with non-union, indicating that each additional millimeter of dislocation increased the odds of non-union. In contrast, implant length was not significantly associated with non-union, independent of medial (OR = 2.77, *p* = 0.342) or lateral (OR = 4.14, *p* = 0.185) dislocation. The model showed improved fit compared to the null model for both medial (null deviance = 91.71, residual deviance = 84.87, AIC = 90.87) and lateral (null deviance = 91.71, residual deviance = 80.1, AIC = 86.1) dislocations as well. The regression model is depicted in Figure 4A,B.

Furthermore, medial and lateral dislocation had a strong trend to an overall increased complication rate (*p* = 0.052). Bony defects due to fracture showed an equally strong trend for implant breakage (*p* = 0.051). The use of cerclage wires was obviously associated with a prolonged time of surgery (102 min vs. 125 min, *p* < 0.01). They did not have any positive or negative impact on overall complication and revision rates in Type B fractures (*p* > 0.05) or in detail on the rates of avascular necrosis as well as mal- or non-union of the fracture (*p* > 0.05). Interestingly, a higher rate of implant failure was found in Type B fractures, which were treated with cerclage wires (*p* = 0.03).

**Figure 5:**
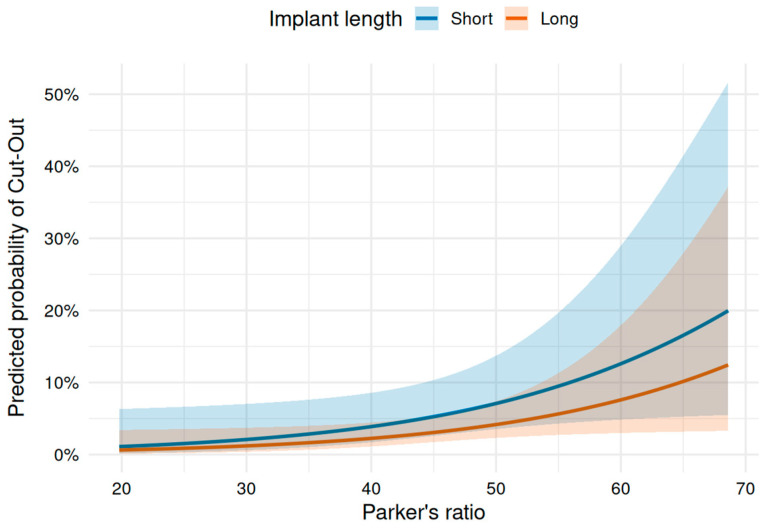
Predictive value of Parker’s ratio on head screw cut-out: A significant predictive value is shown for Parker’s ratio on postoperative cutting-out, independent of choice of implant. In the corresponding figure showing predicted probabilities with 95% confidence intervals (shaded areas), the overlapping shaded bands for long vs. short implants illustrate that the difference between implant lengths is not statistically significant, even though the point estimate lines appear separated.

In our series, only Parker’s ratio showed a significant predictive value for possible screw/blade cut-out (*p* = 0.02), whereas TAD (OR = 1.02, 95% CI: 0.93–1.1, *p* = 0.72) and c-TAD (OR = 1.04, 95% CI: 0.93–1.14, *p* = 0.48) did not. We did logistic regression for Parker’s ratio and implant length as well. Parker’s ratio was significantly associated with cut-out (OR = 1.07, 95% CI: 1–1.13, *p* = 0.0369), indicating that a higher Parker’s ratio increased the odds of cut-out. In contrast, implant length was not significantly associated with cut-out (OR = 0.57, 95% CI: 0.23–1.43, *p* = 0.231). The model showed improved fit compared to the null model (null deviance = 170.78, residual deviance = 164.61, AIC = 170.61). The regression model is depicted in Figure 5.

**Figure 6:**
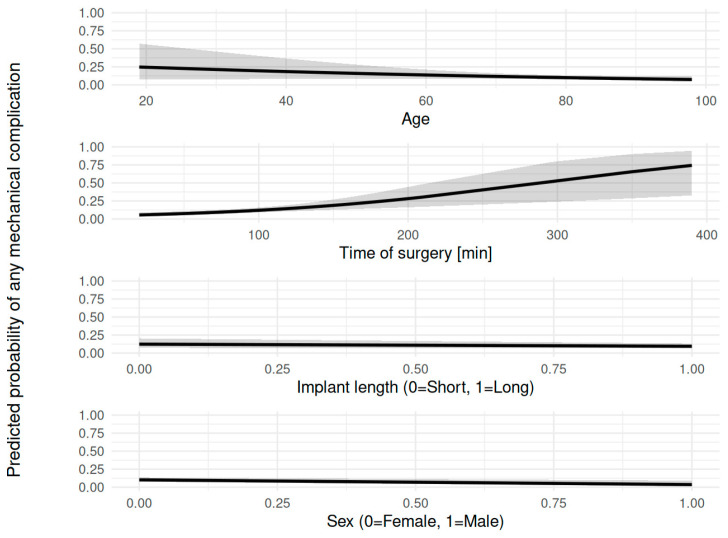
Multivariate analysis of general risk factors on mechanical complications: Prolonged time of surgery and female sex had a significant influence on the mechanical complication rate. Implant length and age did not show significant impact.

**Figure 7:**
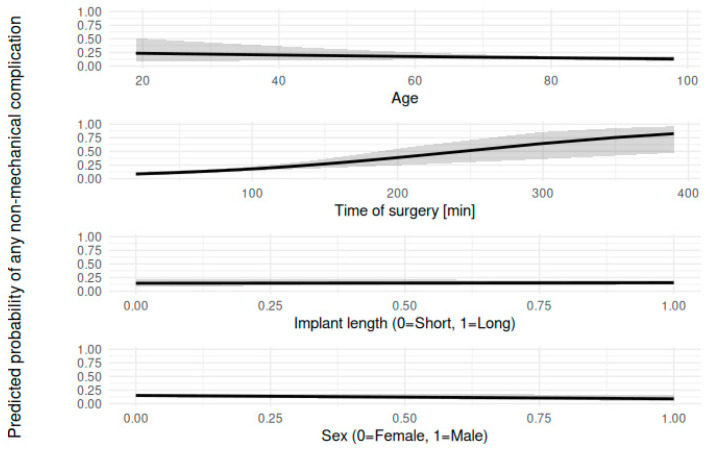
Multivariate analysis of general risk factors on non-mechanical complications: Prolonged time of surgery and female sex had a significant influence on the non-mechanical complication rate. Implant length and age did not show significant impact.

To further minimize risk of selection bias by the surplus of patients treated with long implants, a multivariable logistic regression was conducted to examine the influence of age, time of surgery, implant length, and sex on the overall non-mechanical and mechanical complication rate. Age was 0.99 (OR, 95% CI: 0.97–1.01, *p* = 0.367) for non-mechanical and 0.98 (OR, 95% CI: 0.96–1.01, *p* = 0.13) for the mechanical complication rate. Time of surgery was 1.01 (OR, 95% CI: 1.01–1.02) for non-mechanical and 1.01 (OR, 95% CI: 1–1.02, *p* < 0.001) for the mechanical complication rate, showing a statistically significant increase in the odds of non-mechanical and mechanical complications with longer times of surgery. Implant length indicated no significant effect on non-mechanical 1.07 (OR, 95% CI: 0.57–2, *p* = 0.834) or mechanical 0.74 (OR, 95% CI: 0.36–1.51, *p* = 0.403) complications. Regarding sex, 0.56 for non-mechanical (OR, 95% CI: 0.27–1.16, *p* = 0.119) and 0.33 (OR, 95% CI: 0.12–0.92, *p* = 0.0346) for mechanical complications, the data showed a non-significant trend towards lower non-mechanical complication odds and significantly lower mechanical complication odds for males compared to females. Overall, time of surgery was the only significant predictor for both mechanical and non-mechanical complications, whereas sex had a weaker impact, with males being at lower risk for complications. The other factors seemed not to be associated with the complication rate. The model fit the data reasonably well regarding non-mechanical (AIC = 435.1861115) and mechanical (AIC = 328.2000017) complications. We depicted the multivariate analysis in Figure 6 and Figure 7.

## 4. Discussion

Since reverse-oblique fractures are known to be inherently unstable due to different biomechanical properties [10,11], and therefore associated with increased complication rates, it is reasonable to classify them as a separate sub-entity of trochanteric femoral fractures. A simple and easy-to-use classification system for respective fracture categorization was recently introduced [22]. One of the most controversially discussed questions is certainly whether to use long or short femoral nails for the treatment of these fractures.

The aim of this work was to validate a newly introduced classification system, evaluate its value on clinical decision-making, shed some light on the question of the optimal treatment strategy for reverse-oblique femoral fractures, and look at possible influencing factors on complications and revision surgery.

In the published case series, the fracture pattern did not have a striking statistical influence on the complication rate. The classification is not able to give a definite recommendation regarding the use of long or short cephalomedullary implants, as the failure rates were evenly distributed. The clinical applicability of the classification system is definitely limited. Nevertheless, there was a trend for higher overall complications in complex fracture patterns. Therefore, the use of short implants can be at least safely recommended in fracture types without abruption of the lesser trochanter (Type A) or in fractures without severe dislocation. As we laid great emphasis on this question, we suspect that the fracture pattern should have never been the main factor for the choice of implant as long as the biomechanical requirement of a sufficient implant working length is met. A thorough analysis and a three-dimensional approach to the biomechanical characteristics of these fracture entities would probably be necessary in order to identify a possible connection between fracture pattern and outcome. Due to the high incidence of trochanteric fractures, the cost-effectiveness would be questionable, because the choice of implant seems not to be the main problem in the first place [3,21]. Nevertheless, in this series, with a significant increase in included cases, a good inter- and intra-rater reliability was shown for this classification. Since a reasonable and easy-to-use classification system is currently not available, the introduced classification could serve as a descriptive tool to provide comparability in this highly topical field of research, provide indications of the expected outcome, and aid in decision-making at least in simple fracture patterns.

The complication rate as well as revision rate in our series—at 19% and 11%, respectively—was higher than the 4% commonly found in the literature regarding sole pertrochanteric fractures [28,29]. Even though we included another trauma center in our current study, the acquired numbers were well in line with our previous findings [22]. The stated percentages seem to be accurate in depicting overall complication rates and revision rates in this fracture pattern. But given the high and still-rising incidence of trochanteric femoral fractures, the included numbers may still be too small to analyze particular complications in depth [1]. Nevertheless, these findings underline that reverse-oblique fractures represent a challenging sub-entity of proximal femoral fractures and should, therefore, be treated with caution.

We could not find any predictive value for the introduced classification regarding postoperative complications. This applies to the main types as well as the subtypes A and B, which describe the course of the lateral fracture line. Although the lateral fracture line is taken into consideration, which is a critical parameter for stability [10], the sole a.p. radiograph on which the classification is based seems to be insufficient for describing the complex biomechanics.

The one-year mortality rate in our series was 25%. This certainly is a comparatively high number when compared to proximal femoral fractures in general [5,6], but is well in line with the literature, especially when focusing on unstable intertrochanteric fractures, where rates between 11 and 27% are described [7,21,30]. When compared to the 9% mortality rate within the age-matched general population, the dramatic impact of this injury can be illustrated [7]. Despite the overall high mortality rate, we could not find any influencing factor on mortality beyond the fact of the fracture itself.

Cephalomedullary implants are undisputedly the best available surgical option in the treatment of subtrochanteric femoral fractures [13,31]. However, there is still no consensus regarding cut-off values for implant choice. Currently, simple common recommendations are to treat “proximal” fractures with short nails and “more distal” fractures with long nails [3]. A definition of proximal and distal fractures is lacking. Some authors stress the importance of an intact medial cortex for medial buttress support and suggest a potential higher risk of early catastrophic failure, especially in reverse-oblique fractures [32,33]. A definite benefit from treatment with long implants could not be seen in the previous paper [22]. The literature supports our findings and does not provide evidence for an advantage of long nails in more distal fractures [17,18,19,20]. We placed special emphasis on implant-related complications, but could not find any difference between long and short nails in any of the analyzed parameters. Since we included by far the largest number of patients, we dare to state that the use of long implants as the treatment standard does not lead to any notable benefit in this fracture pattern. In a synopsis of the acquired results and the current literature, a sufficient working length of the implant in relation to the fracture course is the key to avoid implant failure and secondary dislocation [32,33]. Linhart et al. published the only tangible number in this context, and showed that a working length of 2 cm from fracture line to distal interlocking screw is sufficient to guarantee the same stability as a longer construct [3]. However, due to the sole biomechanical analysis, a definite statement cannot be made regarding the clinical value of this information and the absolute minimum in working length has yet to be defined. Regarding the known disadvantages in the use of long implants—like the greater necessity of intramedullary reaming and consecutive systemic inflammatory reaction or the more complicated way of distal locking and connected amplification in time of surgery [33]—we recommend the use of short implants whenever reasonably possible in reverse-oblique fractures, especially in simple Type A fractures. As a limiting factor for these findings, it has to be stated that the majority of patients were treated with a long implant regardless and, therefore, a certain selection bias has to be assumed.

Open reduction posed as a risk factor for overall complication rate. This seems to be a logical consequence of the associated extensive soft tissue damage, damage to local blood supply, and increase in operation time [34,35]. Interestingly, when looking at avascular necrosis or mal- or non-union of the fracture, which are complications commonly associated with soft tissue damage, no special risk could be identified. It may be connected to the low overall numbers of single complications. Nevertheless, the increase in time of surgery was expectedly connected to a higher overall complication rate, and also as a risk factor for the subgroups of mechanical and non-mechanical complications. Furthermore, it was found to be an independent risk factor for a higher infection rate, non-union, and avascular necrosis. These findings support our recommendation to use short implants whenever possible due to the shorter operation time necessary. Of course, an increase in time of surgery is often derived from more complicated intra-operative situations, either regarding fracture complexity or the necessity of open reduction and the use of cerclage wires.

As another risk factor, the patient’s sex was identified, where males had an advantage. Proximal femoral fractures of any kind are known to be predominant in women, due to the higher incidence of osteoporosis [26,27]. Consequently, reduced bone quality in females seems to have an especially negative influence on developing mechanical complications.

Futamura et al. emphasized the importance of an intact lateral cortex for fracture stability, whereas Viberg et al. felt convinced that an intact medial cortex provides the necessary buttress support [10,33]. Inadequate reduction and implant placement have been mentioned as risk factors for catastrophic failure [7,35]. In our series, medial and lateral malreduction were found as independent risk factors for increased overall complication rates and a particularly higher risk for mal- or non-union of the fracture and consequent implant failure. Notably, the risk of implant failure was enhanced in the case of a bony defect, where fracture healing was delayed because of the necessity of bone bridging the gap and, therefore, the implants’ load cycles are surpassed. This, in turn, underlines the inherent instability in this fracture pattern and the necessity of careful anatomical reduction.

The use of cerclage wires seems to be a tempting opportunity to reach the desired reduction, but in fact should be utilized with caution. As already stated, open reduction intensifies the occurrence of complications and definitely increases time of surgery, which in turn causes further problems. Nevertheless, the recent literature recommends the use of cerclage wires in cases when open reduction has to be performed anyway [34,35]. In our series, cerclage wires had no positive effect on any particular complications or revision rates. On the contrary, a higher rate of implant failure was found in patients treated with cerclage wires. We suspect that delayed healing had to be expected in these cases from the beginning because the need for open reduction and application of cerclages indicate a more complex situation. Nevertheless, the disruption of bony blood supply due to soft tissue damage in open reduction can play an important role as well. Maybe minimally invasive application techniques, which were not performed in our collective, potentially provide better results overall [34,35]. Regarding an unavoidable increase in time of surgery and the above-stated risk of a higher complication rate, open reduction and the application of cerclage wires should nevertheless be limited to cases where an adequate and sufficient reduction is definitely not possible. However, as already mentioned as well, the complexity of the fracture pattern and the consequent intra-operative situation have to be considered as well.

Established radiological prediction parameters repeatedly had only mild prognostic value for the risk of cut-out in our series, since only Parker’s ratio showed any correlation with increased implant failure. This correlates with the current literature regarding reverse-oblique fracture types [28,35]. Initially, this may lead to the assumption that the different biomechanical properties induce an increase in other major modes of failure. The findings in our series are contrary to this hypothesis, since the most frequent mechanical complication was screw cut-out. To our knowledge, no biomechanical study exists to provide a decent explanation for these results, so we can only theorize about possible reasons. With age, the femoral neck develops a varus configuration [36]. Maybe the lack of support around the calcar region in combination with the commonly poor bone quality due to osteopenic or osteoporotic changes and increased shear forces—which have to be absorbed by the nail—creates a combination of factors enabling the occurrence of cut-outs even when the femoral neck screws or blades are positioned correctly. However, the findings have a certain impact on the clinical practice and intra-operative evaluation of an acceptable radiological result. The correct implant position is certainly an important factor, but we recommend to emphasize the proper fracture reduction, as it seems to be of higher prognostic value.

There are some limitations to this study. First of all, the retrospective study design. Most of the patients were treated with long implants, which hampers comparability at least for Type B fractures. To counter possible selection bias, regression models were conducted, correcting for implant length if the results were statistically significant. The choice of implant and the use of cerclage wires was an individual decision by the surgeon. A sole a.p. radiograph seems insufficient to describe the complex biomechanics of reverse-oblique fractures, which limits the clinical value of the introduced classification.

The strengths of this study include the large overall number of included cases, which enables sufficient statistical power for the conducted analyses. To the best of our knowledge, this case series represents the largest number of patients included with a sole focus on the reverse-oblique fracture pattern. Additionally, the investigation of particular complications in relation to influencing factors was possible. Due to the association of these injuries with age and osteoporosis, an epidemiologically homogeneous patient collective could be included.

## 5. Conclusions

The used classification system has an acceptable inter- and intra-rater reliability and can be useful to guarantee comparability in a challenging field of research. As a tool for clinical decision-making, a classification based on simple radiographs seems to be of limited use. Nevertheless, the use of short implants can be safely recommended in Type A fractures and fracture patterns without severe dislocation. In these cases, a good outcome can be expected. There is no indication that long implants guarantee any overall advantage in the treatment of reverse-oblique femoral fractures. Short implants should be used whenever possible to shorten time of surgery, but a sufficient working length is mandatory. In reverse-oblique trochanteric fractures, a high complication and mortality rate is present. Anatomical reduction seems to be of great importance. Closed reduction should be performed whenever possible. The use of cerclage wires remains controversial because of potentially better fracture reduction, although the prolonged time of surgery needs to be acknowledged. More high-quality biomechanical and clinical studies have to be conducted to answer the question of implant length, to better understand failure mechanisms and, as a consequence, prevent complications. Bigger case series are necessary to examine influencing factors on particular complications. An easy-to-use classification system to facilitate the choice of treatment, as well as delivering prognostic value, is still lacking.

## Figures and Tables

**Table 1:** Overview of fracture pattern distribution and used implants.

Fracture Type	Long Implant	Short Implant	Total
I	52	38	90
a	22	31	53
b	30	7	37
II	180	57	237
a	92	44	136
b	88	13	101
III	24	26	50
a	14	22	36
b	10	4	14
IV	132	43	175
a	69	40	109
b	63	3	66

**Table 2:** Overview of complications and revision surgery.

Complications	Absolute Number	Percentage
Necessary Revision Surgery	65	11%
Cut-Out	21	3%
Implant Failure	18	3%
Pseudarthrosis	9	2%
Avascular Necrosis	6	1%
Deep Infection	15	3%
Implant Loosening	8	1%
Lateral Screw/Blade Migration	10	2%
Minor Complications	18	3%
Total	105	19%

## Data Availability

The raw data supporting the conclusions of this article will be made available by the authors upon request.
